# Oxygen-Sparing Anesthesia with Electrically Controlled Ventilators: A Bench Study with Implications for Clinical Practice and Resource Management

**DOI:** 10.1213/ANE.0000000000007270

**Published:** 2024-09-13

**Authors:** Vito Torrano, Francesco Zadek, Giacomo Abbiati, Chiara Deli, Roberto Fumagalli, Thomas Langer

**Affiliations:** From the * Department of Anesthesia and Intensive Care Medicine, Niguarda Ca’ Granda, Milan, Italy; † Department of Medicine and Surgery, University of Milan-Bicocca, Monza, Italy.

The coronavirus disease-2019 (COVID-19) crisis exposed critical oxygen shortages globally, especially in low- and middle-income countries. The World Health Organization recently passed a resolution to conserve medical oxygen due to its impact on patient care.^1^ Modern anesthesia machines use both oxygen and medical air to function. The aim of this study was to analyze the oxygen consumption of several anesthesia ventilators applying different ventilatory settings, with the goal of understanding how oxygen is used during general anesthesia.

## MATERIALS

The study was conducted at the Grande Ospedale Metropolitano Niguarda in Milan, Italy. Four different anesthesia workstations were compared: Dräger Primus, Dräger Atlan, Getinge Flow-i, and Mindray A9.

**Table.** Oxygen Consumption of Each Ventilator Measured at Different Fio_2_, Set Fresh Gas Flows, and Ventilatory Settings

Ventilator	FiO_2_ %	Oxygen consumption L/min					
		TV 500 mL, RR 12/min			TV 250 mL, RR 24/min		
		Fresh gas flow			Fresh gas flow		
		1 L/min	7 L/min	15 L/min	1 L/min	7 L/min	15 L/min
Dräger Primus	30	0.2 (0.0)	1.2 (1.0)	2.3 (0.6)	0.1 (0.6)	1.1 (0.0)	2.2 (1.0)
	50	0.4 (0.0)	2.8 (0.6)	5.9 (0.0)	0.4 (0.6)	2.8 (0.6)	5.7 (1.5)
	70	0.7 (1.4)	4.5 (0.6)	9.5 (0.6)	0.6 (0.6)	4.4 (0.0)	9.2 (0.6)
Dräger Atlan	30	0.2 (0.0)	1.0 (1.0)	2.1 (0.6)	0.2 (0.6)	1.0 (0.0)	2.2 (1.5)
	50	0.4 (0.6)	2.7 (1.0)	5.8 (1.5)	0.4 (0.6)	2.8 (0.6)	5.8 (0.6)
	70	0.7 (0.6)	4.6 (0.6)	9.8 (0.6)	0.7 (1.0)	4.5 (1.0)	9.7 (0.6)
Mindray A9	30	12.9 (0.6)	11.6 (1.0)	9.4 (0.0)	13.8 (0.6)	12.4 (0.6)	10.1 (1.0)
	50	13.1 (0.0)	13.1 (0.6)	13.0 (0.6)	13.7 (0.0)	13.3 (1.0)	13.3 (0.0)
	70	13.3 (0.0)	14.8 (0.6)	16.9 (1.5)	14.3 (0.0)	15.9 (0.0)	17.4 (0.6)
Getinge Flow-i	30	6.3 (1.0)	2.2 (0.6)	2.1 (0.6)	6.9 (0.6)	2.6 (0.6)	2.6 (0.6)
	50	7.1 (1.5)	3.7 (0.6)	3.7 (0.0)	7.8 (0.6)	4.0 (0.6)	4.0 (0.6)
	70	6.7 (0.6)	4.8 (0.6)	4.8 (0.0)	7.4 (0.0)	5.4 (0.6)	5.5 (0.6)

Oxygen consumption is expressed in L/min and refers to pure oxygen consumed, that is, it does not include oxygen deriving from medical air. Data are presented as mean (SD).

Abbreviations: FiO_2_, fraction of inspired oxygen, expressed as %; RR, respiratory rate; SD, standard deviation; TV, tidal volume.

**Figure.**
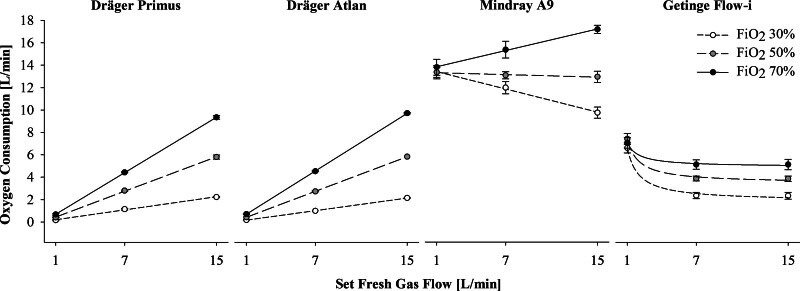
Relationship between pure oxygen consumption and set fresh gas flow in the different anesthesia ventilators. Oxygen consumption is expressed in L/min and refers to pure oxygen consumed, that is, it does not include oxygen deriving from medical air. White dots represent tests performed at FiO_2_ of 30%. Light gray dots represent tests performed at FiO_2_ of 50%. Black dots represent tests performed at FiO_2_ of 70%. Data are presented as mean ± standard deviations and represent the average value of oxygen consumption obtained by pooling together the data from the 2 combinations of tidal volume and respiratory rate. FiO_2_ indicates fraction of inspired oxygen, expressed as %.

Each ventilator underwent testing with 18 unique combinations of tidal volume and respiratory rate (500 mL × 12/min vs 250 mL × 24/min), fresh gas flow (FGF) (1, 7, and 15 L/min), and a fraction of inspired oxygen (FiO_2_; 30%, 50%, and 70%), using a simple lung ventilator without oxygen consumption or carbon dioxide production. Parameters were kept constant for 30 minutes, and the volume of pure medical oxygen consumed was measured using a Mass Flowmeter (MF5000, Siargo Ltd) connected in series to the oxygen supply line between the wall pipeline and the ventilator. All experiments were repeated in triplets.

## RESULTS

Medical oxygen consumed for the performance of the whole set of combinations, which lasted 9 hours, differed between anesthesia ventilators: 1622 and 1638 L for Primus and Atlan, respectively; 7249 L for A9, and 2630 L for Flow-i. The Table summarizes the consumption of medical oxygen in different ventilatory settings. A remarkable difference in oxygen consumption was observed among ventilators for the different FGF and FiO_2_ combinations (Figure). In particular, Primus and Atlan had a linear increase in oxygen consumption according to the applied FGF and FiO_2_, while A9 and Flow-i ventilators exhibited different and less predictable behaviors.

## DISCUSSION

The observed differences in oxygen consumption are justified by the distinct operational mechanisms of the used ventilators. Dräger ventilators do not require a driving gas, as an electric mechanism propels gases using a bellow or piston.^2,3^ Conversely, for Flow-i and A9 ventilators, oxygen plays a dual role, serving both as “inspired” and as “driving” gas.^4^ Consequently, these ventilators consume larger volumes of oxygen when operating at low FGFs and FiO_2_. In a scenario of absence of pressurized oxygen, electrically driven machines, and some driving-gas-dependent ventilators remain functional, albeit ventilating with room air. On the contrary, if also compressed medical air is lacking, only electrically driven ventilators are able to guarantee mechanical ventilation at room air. Of note, both types of ventilators require electricity to function.

Despite using similar driving-gas technologies, Flow-i and A9 had different oxygen consumptions at increasing FGFs. Notably, at FGF exceeding minute ventilation, Flow-i had a relatively low oxygen consumption, which did not change with FGF. This information might suggest the ability to automatically limit FGF, thus avoiding an unnecessary waste of oxygen.

Overall, sparing oxygen might have significant economic implications and clinical relevance in low- and middle-income countries (LMICs) and in scenarios of limited oxygen resources, such as during wartime.^5^ Recently, pressure swing adsorption (PSA) on-site plants are finding increasing acceptance in supplying medical oxygen. While this strategy has potentially a lower environmental impact, PSA has a fixed production capacity.^6^ Taking into consideration the impact of anesthesia machines on oxygen consumption is thus fundamental to properly plan on-site PSA plants. However, in many LMICs health care facilities cylinders still constitute the primary source of oxygen supply, and oxygen delivery might thus be subject to interruptions.

Based on our data, electronically-driven ventilators are a valid option in this context. If, for example, the oxygen source should be switched to a standard 15-L cylinder filled at 200 atm, Primus ventilator would allow 250 hours of ventilation at an FiO_2_ of 30% with an FGF of 1 L/min, while only 4 hours of general anesthesia could be performed with the A9 ventilator. The logistical advantage is self-explanatory.

Lastly, a recent study estimated the environmental impact of 1 Nm^3^ of medical oxygen to be 256 kg of CO_2_ when provided through a liquid oxygen tank and up to 555 kg when provided in cylinders.^7,8^ Assuming the surgical activity of a large hospital with 30 operating rooms, performing 10 hours of general anesthesia 20 days each month with a FiO_2_ of 50% and an FGF of 1 L/min, we can calculate a rough difference in oxygen consumption between the most and the least efficient ventilator of approximately 55,000 m^3^ of oxygen per year, corresponding to 14,000 tons of CO_2_. However, the complete economic and environmental impact of anesthesia machines should encompass both volatile anesthetics^9^ and electrical consumption.^10^

It is important to underline that results obtained at low FGF are the most relevant as the higher FGF are rarely used for the maintenance of anesthesia. During general anesthesia, electrically-driven ventilators allow significant oxygen savings, being a preferable option in contexts where oxygen supply is discontinuous.

## DISCLOSURES

**Conflicts of Interest:** None. **Funding:** None. **This manuscript was handled by:** Thomas M Hemmerling, MSc, MD, DEAA.

## References

[1] World Health Organization (WHO). Increasing access to medical oxygen. Seventy-sixth World Health Assembly. May 30, 2023. Accessed September 11, 2024. https://apps.who.int/gb/ebwha/pdf_files/WHA76/A76_R3-en.pdf

[2] Dräger Medical GmbH. Instructions for use Primus Anesthesia workstation SW 4.5n. 2015. Accessed September 11, 2024. https://www.draeger.com/Content/Documents/Content/IfU_Primus_SW_4.5n_EN_9053461.pdf

[3] Dräger Medical GmbH. Instructions for use Atlan A300, A300 XL, A350, A350 XL. 2019. Accessed September 11, 2024. https://www.draeger.com/Content/Documents/Content/atlan-a300-a300-xl-a350-a350-xl-ifu-9056001-en.pdf

[4] Mindray DS USA, Inc. Operating instruction A9 anesthesia system. January 4, 2024. Accessed September 11, 2024. https://www.mindray.com/etc.clientlibs/xpace/clientlibs/clientlib-site/resources/plugins/web/viewer.html?file=/content/dam/xpace/en_us/products-solutions/products/anesthesia/a-series-work-station/technical-documents/H-046-017199-00-A9-Operators-ManualFDA.pdf

[5] Szpisjak DF Starrett-Keller CM. Field anesthesia machine ventilator oxygen consumption in models of high and low pulmonary compliance. Mil Med. 2014;179:1465–1468. 25469969 10.7205/MILMED-D-14-00205

[6] Tariq M Siddhantakar A Sherman JD Cimprich A Young SB. Life cycle assessment of medical oxygen. J Clean Prod. 2024;444:141126.

[7] Bałys M Brodawka E Korzeniewska A Szczurowski J Zarębska K. LCA and economic study on the local oxygen supply in Central Europe during the COVID-19 pandemic. Sci Total Environ. 2021;786:147401. 33964772 10.1016/j.scitotenv.2021.147401 PMC8081744

[8] Seglenieks R McAlister S McGain F. Environmental impact of medical oxygen production in Australia. Comment on Br J Anaesth 2020; 125: 773–8. Br J Anaesth. 2021;127:e104–e105. 34275602 10.1016/j.bja.2021.06.028

[9] Varughese S Ahmed R. Environmental and occupational considerations of anesthesia: A narrative review and update. Anesth Analg. 2021;133:826–835. 33857027 10.1213/ANE.0000000000005504 PMC8415729

[10] Drinhaus H Drinhaus J Schumacher C Schramm MJ Wetsch WA. Electricity consumption of anesthesia workstations and potential emission savings by avoiding standby. Anaesthesiologie. 2024;73:244–250. 38349537 10.1007/s00101-024-01388-3 PMC11021308

